# Risk and Prognostic Factors for Multidrug-Resistant *Acinetobacter Baumannii* Complex Bacteremia: A Retrospective Study in a Tertiary Hospital of West China

**DOI:** 10.1371/journal.pone.0130701

**Published:** 2015-06-17

**Authors:** Qianqian Liu, Wenzhang Li, Xinmiao Du, Weijing Li, Taiqing Zhong, Yin Tang, Yulin Feng, Chuanmin Tao, Yi Xie

**Affiliations:** 1 Department of Laboratory Medicine, West China Hospital, Sichuan University, Chengdu, Sichuan, China; 2 Department of Cardiology, First Affiliated Hospital of Chengdu Medical College, Chengdu, Sichuan, China; 3 Department of Respiratory Medicine, West China Hospital, Sichuan University, Chengdu, Sichuan, China; 4 Division of Oral Biology, Tufts University School of Dental Medicine, Boston, Massachusetts, United States of America; Curtin University, AUSTRALIA

## Abstract

**Background:**

The increasing prevalence and mortality of multidrug-resistant (MDR) *Acinetobacter baumannii* complex-associated infections, especially bacteremia, in health care settings poses a great threat to public health. We proceeded to investigate the risk and prognostic factors for MDR *A*. *baumannii* complex bacteremia in mainland China.

**Methods:**

This retrospective study was conducted at West China Hospital from January 2009 to December 2013. Using a computer-assisted microbiology laboratory database, patients with MDR *A*. *baumannii* complex bacteremia were included as the case group, while those infected with non-MDR *A*. *baumannii* complex were selected as the control group. The clinical data were collected and analyzed.

**Results:**

There were 241 non-duplicated *A*. *baumannii* complex blood isolates identified in our research, with the overall rate of multidrug resistance reaching 75.52% over the past five years. Using multivariate logistic analysis, being in the intensive care unit (ICU) (adjusted odds ratio [aOR], 5.84; 95% confidence interval [CI], 1.67-20.44), increased Pittsburgh bacteremia score (aOR, 6.55; 95% CI, 1.27-33.70) and use of carbapenem (aOR, 8.90; 95% CI, 1.71-46.30) were independent risk factors for MDR acquisition among patients with *A*. *baumannii* complex bacteremia. Older age (aOR, 1.02; 95% CI, 1.00-1.04), being post-transplantation (aOR, 5.21; 95% CI, 1.13-24.04), having a higher Pittsburgh bacteremia score (aOR, 2.19; 95% CI, 1.08-4.47) and having a lower level of albumin (aOR, 0.93; 95% CI, 0.88-0.99) were identified as independent risk factors for 30-day mortality in patients with MDR *A*. *baumannii* complex bacteremia.

**Conclusion:**

In conclusion, our research revealed the risk factors associated with acquisition of and mortality from MDR *A*. *baumannii* complex bacteremia, which may be used to prioritize infection control practices and prognostic evaluations.

## Introduction

*Acinetobacter baumannii*, a non-fermentative Gram-negative coccobacillus, has become an increasingly notorious pathogen in health care settings due to its enhanced environmental resilience [1]. It is estimated that *A*. *baumannii* comprises 4% to 7% of ventilator-associated pneumonia and 1% to 2% of nosocomial bloodstream infections [2–5]. Based on epidemiological studies, the mortality rates of infections associated with *A*. *baumannii* have reached a range from 10% to 43% in intensive care units (ICUs) and from 7.8% to 23% outside ICUs, which significantly increased the outlay of the infirmary [6].

It is generally recognized that the propensity for antimicrobial resistance in *A*. *baumannii* usually leads to a high rate of treatment failure, and the clinically-isolated multidrug-resistant (MDR) *A*. *baumannii* also tend to be increasing over time [7–9]. Although *A*. *baumannii* can invade different parts of the body, such as the respiratory tract, bloodstream, or skin and soft tissue, MDR *A*. *baumannii* bacteremia usually leads to especially high attributable mortality [10]. Accordingly, investigating the clinical characteristics and influencing factors of MDR *A*. *baumannii* bacteremia has become extremely urgent.

However, until recently, few retrospective studies have evaluated the factors influencing drug-resistance acquisition in *A*. *baumannii* bacteremia and its outcomes [11–13]. Several studies used patients without *A*. *baumannii* as controls to identify risk factors only for *A*. *baumannii* bacteremia [13]. Beyond that, other possible risk factors were not included in these analyses, and results from hospitals in different geographic locations may differ due to variations in infection control implementation, antibiotics resources, and the disease spectrum. Thus, we proceeded to investigate the risk and prognostic factors for MDR *A*. *baumannii* complex bacteremia in mainland China. This study was performed in West China Hospital, a 4,000-bed tertiary hospital in Chengdu, southwest China, using our computer-assisted microbiology laboratory database.

## Materials and Methods

### Study population and design

This retrospective study was conducted at West China Hospital from January 2009 to December 2013. Using the microbiology laboratory records, patients aged > = 18 years, with at least one blood culture positive for *A*. *baumannii* complex were selected, and their clinical data were obtained from the electronic medical records for review. For patients with multiple episodes of *A*. *baumannii* complex bacteremia, only the first episode was included for analysis. Those included patients were further divided into cases or controls according to antibiotics susceptibility results. The case group was comprised of patients with MDR *A*. *baumannii* complex bacteremia, and the control group included patients with any other *A*. *baumannii* complex bacteremia.

### Ethics statement

This study was approved by the ethics review committee of West China Hospital, Sichuan University, Chengdu, China, which waived informed consent.

### Microbiology methods

During the study period, blood specimens were obtained under sterile conditions and cultured using the BacT/Alert 3D blood culture system (bioMerieux, Durham, USA). Identification of microorganisms was conducted using the VITEK 2 compact (bioMerieux, Durham, USA), and *A*. *baumannii* complex described here included the following 4 species: *A*. *baumannii*, *A*. *nosocomialis*, *A*. *pittii*, and *A*. *calcoaceticus*, which cannot been distinguished by conventional biochemical methods [14,15]. Susceptibility testing of Cefperazone/sulbactam was performed using the manual disk diffusion method. For other antimicrobials, susceptibility testing was conducted using the VITEK 2 compact (bioMerieux, Durham, USA). The susceptibility results were interpreted following the breakpoints defined by the most current Clinical and Laboratory Standards Institute [16]. Multidrug resistance was defined as non-susceptibility to 3 or more of the following groups of antimicrobials: aminoglycosides, anti-pseudomonal penicillins, carbapenems, cephalosporins, and quinolones [17].

### Data collection

A standardized case report form was designed to collect the relevant demographic, clinical, and laboratory data following medical record review. The following details were gathered: age, gender, body mass index (BMI), underlying diseases, primary admission diagnosis, sources of bacteremia, Pittsburgh bacteremia score [18], duration of hospital stay and ICU stay before the development of bacteremia, invasive therapy (chemotherapy or radiotherapy), treatment with cytotoxic drugs or corticosteroids (≥20 mg/day for >5 days) that occurred within 14 days before the onset of bacteremia, invasive devices (surgery, dialysis, and invasive devices including, central venous catheter, nasogastric tube, urinary catheter, drain, ventilation), surgical procedure within the past 14 days, hospitalization prior to development of bacteremia within the past three months, antibiotic therapy before (≥5 days) the onset of bacteremia, outcomes of patients with *A*. *baumannii* complex bacteremia, the sample date and report date of the first positive blood culture for *A*. *baumannii* complex, co-infection with other pathogens in the bloodstream, antibiotic susceptibility results, and the level of serum albumin.

### Definition

The criteria for infection diagnosis were based upon National Healthcare Safety Network criteria [19]. Bacteremia was diagnosed if a pathogen was isolated from one or more blood samples and the patient had clinical symptoms and signs of infection. The sources of bacteremia were assessed by study investigators according to clinical signs, symptoms, image data, surgical findings, and microbiological evidence. They were categorized into respiratory infection, urinary tract infection, biliary tract infection, catheter-related bloodstream infection (CRBSI), post-surgical wound infection, central nervous system infection and intra-abdominal infection. The severity of illness was estimated by a grading system, the Pittsburg bacteremia score, on the date of index culture [18].

### Statistical analysis

Continuous variables were compared using the student *t* test or the Mann-Whitney U test as appropriate. Categorical variables were calculated using a chi-square test or Fisher’s exact test as appropriate. The trend of proportion of isolates that were MDR was assessed using a linear by linear association test. Univariate analysis was used to identify significant factors for MDR acquisition and mortality, with results presented as odds ratios (OR) and 95% confidence intervals (95% CI). Independent covariates with a *p* value <0.10 were included in a multivariate logistic regression analysis. The survival curve was generated using the Kaplan-Meier method, and the log-rank test was used to compare survival between groups. All tests of significance were 2-sided, and significance was set at *p* value <0.05. Statistical analyses were performed using SPSS software (version 18.0, SPSS Inc, Chicago, IL, USA).

## Results

### *In vitro* antibiotic susceptibility tests and distribution of *A*. *baumannii* complex isolates

**Fig 1:**
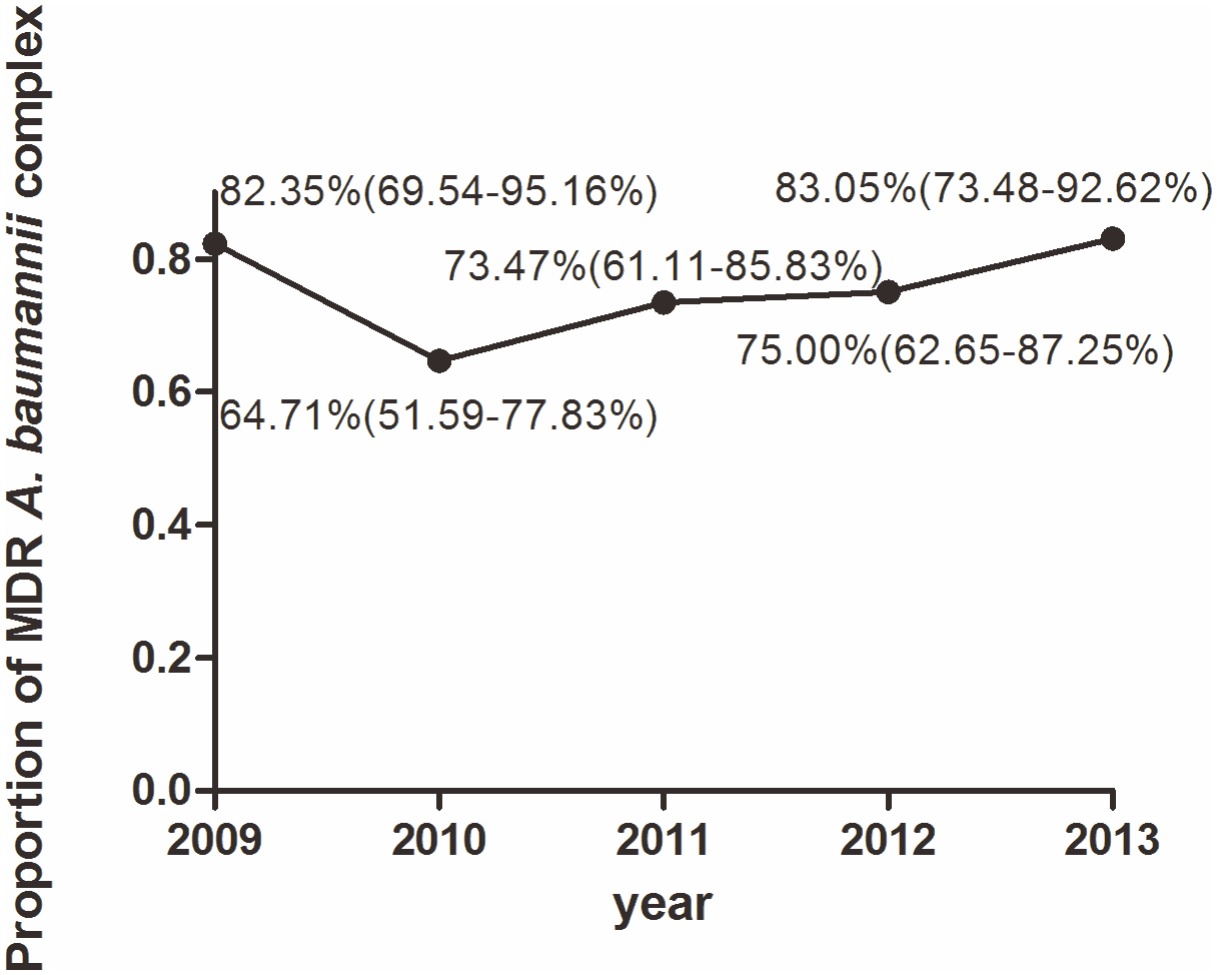
The annual trend in the MDR rate in *A*. *baumannii* complex bacteremia from January 2009 to December 2013.

We identified 241 non-duplicated *A*. *baumannii* complex blood isolates between January 2009 and December 2013, including 182 MDR *A*. *baumannii* complex blood isolates. As shown in Fig 1, the MDR rate decreased from 2009 to 2010 and then rose (*p* = 0.032) from 2010 to 2013. The susceptibilities of *A*. *baumannii* complex isolates to 15 types of routinely reported antibiotics in the MDR group and the non-MDR group were showed in S1 Table. It is worth noting that MDR *A*. *baumannii* complex was highly resistant to all antibiotics (>90%) except amikacin (52.0%), tobramycin (87.4%) and trimethoprim-sulfamethoxazole (69.3%). However, non-MDR *A*. *baumannii* complex exhibited low resistance to all antibiotics (<50%), with the exceptions of cefotaxime (61.9%), ceftriaxone (71.2%) and aztreonam (93.1%).

**Fig 2:**
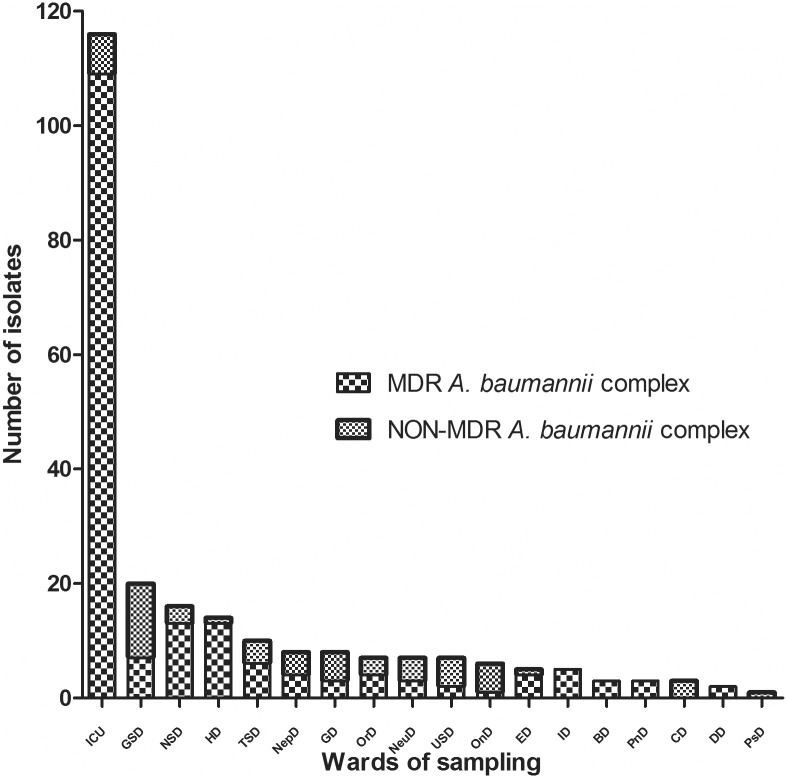
The distribution of *A*. *baumannii* complex isolates from blood samples with their MDR constituent ratios across different hospital department. ICU, intensive care unit; GSD, general surgery department; NSD, neurosurgery department; HD, hematological department; TSD, thoracic neurosurgery department; NepD, nephrological department; GD, gastroenterological department; OrD, orthopedics department; NeuD, neurological department; USD, urinary neurosurgery department; OnD, oncological department; ED, emergency department; ID, infectious department; BD, burn department; PnD, pneumological department; CD, cardiological department; DD, dermatological department; PsD, psychiatry department.

The distribution of *A*. *baumannii* complex isolates from blood sample with their MDR constituent ratios across different hospital department are shown in Fig 2. Among all wards, the ICU was the main source of both *A*. *baumannii* complex (48.1%) and MDR *A*. *baumannii* complex (59.9%), followed by two surgical departments (general surgery department and neurosurgery department) and the hematological department.

### Risk factors and outcomes for MDR *A*. *baumannii* complex bacteremia

There were no differences in gender, BMI, prior invasive therapy or prior surgical procedures between the patients with MDR *A*. *baumannii* complex bacteremia and non-MDR *A*. *baumannii* complex bacteremia. By univariate logistic analysis (S2 Table), patients admitted to the hospital for acute pancreatitis, those with respiratory route or post-surgical wounds as the source of bacteremia, those with prior use of invasive devices, those with prior hospitalization, those with previous use of glucocorticoids or immunosuppressors, and those in the ICU were significantly more likely to have acquired MDR *A*. *baumannii* complex. Patients with solid-organ malignancy showed reduced risk of MDR *A*. *baumannii* complex bacteremia. In addition, prior exposure to carbapenem, piperacillin-tazobactam, cefperazone-sulbactam, floroquinolones, vancomycin, and antifungal drugs were more common among the MDR *A*. *baumannii* complex group. Those patients with MDR *A*. *baumannii* complex bacteremia also tended to be younger, to have more severe conditions. By multivariate logistic analysis (Table 1), staying in the ICU (aOR, 5.84; 95% CI, 1.67–20.44), increased Pittsburgh bacteremia scores (aOR, 6.55; 95% CI, 1.27–33.70) and prior use of carbapenem (aOR, 8.90; 95% CI, 1.71–46.30) were independently associated with MDR *A*. *baumannii* complex bacteremia. The 30-day cumulative survival rate showed that patients with MDR *A*. *baumannii* complex bacteremia had a significantly lower survival rate than those with non-MDR *A*. *baumannii* complex bacteremia (70.9% vs. 96.6%, p<0.001 using the log-rank test).

**Table 1:** Comparison of demographics, clinical characteristics, infection status, and outcome of patients with MDR *A*. *baumannii* complex bacteremia and non-MDR *A*. *baumannii* complex bacteremia using a multivariate model.

Variables	MDR *A*. *baumannii* complex	Non-MDR *A*. *baumannii* complex	Multivariate analysis		
	n = 182	n = 59	Adjusted OR	95% CI	*p*-value
**Age, mean±SD (y)**	52.423±18.507	58.678±15.117	-	-	0.100
**Underlying disease of solid-organ malignancy, n (%)**	20 (11.0%)	13 (22.0%)	-	-	0.639
**Acute pancreatitis as primary admission diagnosis, n (%)**	41 (22.5%)	6 (10.2%)	-	-	0.428
**Respiratory infection as sources of bacteremia, n (%)**	130 (71.4%)	21 (35.6%)	-	-	0.231
**Post-surgical wound infection as sources of bacteremia, n (%)**	12 (6.6%)	0 (0.0%)	-	-	0.993
**Pittsburgh score≥4, n (%)**	59 (32.4%)	2 (3.4%)	6.55	1.27–33.70	0.025
**Presence of invasive devices, n (%)**	167 (91.8%)	38 (64.4%)	-	-	0.345
**Hospitalization within the past three months, n (%)**	111 (61.0%)	21 (35.6%)	-	-	0.108
**Stay in ICU, n (%)**	109 (59.9%)	7 (11.9%)	5.84	1.67–20.44	0.006
**Glucocorticoids/immunosuppressor use within the past 14 days, n (%)**	74 (40.7%)	6 (10.2%)	-	-	0.451
**Piperacillin/tazobactam use within the past 14 days, n (%)**	51 (28.0%)	6 (10.2%)	-	-	0.844
**Cefoperazone/sulbactam use within the past 14 days, n (%)**	47 (25.8%)	6 (10.2%)	-	-	0.263
**Carbapenems use within the past 14 days, n (%)**	88 (48.4%)	2 (3.4%)	8.90	1.71–46.30	0.009
**Floroquinolones use within the past 14 days, n (%)**	43 (23.6%)	5 (8.5%)	-	-	0.510
**Vancomycin use within the past 14 days, n (%)**	44 (24.2%)	2 (3.4%)	-	-	0.308
**Anti-fungal agents use within the past 14 days, n (%)**	45 (24.7%)	3 (5.1%)	-	-	0.850

MDR, multidrug-resistant; SD, standard deviation; OR, odds ratio; CI, confidence interval; ICU, intensive care unit.

### Risk factors for 30-day mortality in patients with MDR *A*. *baumannii* complex bacteremia

Overall, no significant differences in gender, BMI, culture detection time, imipenem resistance, primary admission diagnosis, invasive therapy, ICU stay, and simultaneous infection with another pathogen were observed between the two groups of patients. In univariate analysis for 30-day mortality (S3 Table), older patients, those with chronic pulmonary disease, those admitted for acute pancreatitis, those who were post-transplantation, those with high Pittsburgh bacteremia score, those complicated with *Enterococcus faecium* bacteremia and those with lower levels of albumin were associated with poorer prognosis. Older age (aOR, 1.02; 95% CI, 1.00–1.04), being post-transplantation (aOR, 5.21; 95% CI, 1.13–24.04), high Pittsburgh bacteremia score (aOR, 2.19; 95% CI, 1.08–4.47) and lower level of albumin (aOR, 0.93; 95% CI, 0.88–0.99) remained independent risk factors for 30-day mortality in patients with MDR *A*. *baumannii* complex bacteremia in the multivariate analysis (Table 2).

**Table 2:** Comparison of risk factors associated with mortality in MDR *A*. *baumannii* complex bacteremia using a multivariate model.

Variables	Mortality	Survival	Multivariate analysis		
	n = 53	n = 129	Adjusted OR	95% CI	*p*-value
**Age, mean±SD (y)**	56.434±18.510	50.775±18.324	1.02	1.00–1.04	0.041
**Underlying disease of chronic pulmonary disease, n (%)**	10 (18.9%)	12 (9.3%)	-	-	0.383
**Underlying disease of post-transplantation, n (%)**	5 (9.4%)	3 (2.3%)	5.21	1.13–24.04	0.034
**Acute pancreatitis as primary admission diagnosis, n (%)**	6 (11.3%)	35 (27.1%)	-	-	0.094
**Pittsburgh bacteremia score ≥4, n (%)**	23 (43.4%)	36 (27.9%)	2.19	1.08–4.47	0.031
**Complicated with *Enterococcus faecium* bacteremia**	8 (15.1%)	9 (7.0%)	-	-	0.067
**Serum albumin (g/L)**	28.059±7.569	31.953±12.063	0.93	0.88–0.99	0.017

MDR, multidrug-resistant; SD, standard deviation; OR, odds ratio; CI, confidence interval.

## Discussion

*A*. *baumannii* complex, which has a great propensity for epidemic spread, has emerged as a common pathogen of health care-associated infections and poses a growing threat to public health [20]. An even more serious situation is the increasing rate of MDR among *A*. *baumannii* complex, which usually leads to high treatment failure [7]. Thus we aimed to investigate the risk and prognostic factors for MDR *A*. *baumannii* complex bacteremia in mainland China. In total, 241 patients with *A*. *baumannii* complex blood isolates were included in our research. Staying in the ICU, increased Pittsburgh bacteremia score and prior use of carbapenem were independent risk factors associated with MDR *A*. *baumannii* complex bacteremia. Patients with MDR *A*. *baumannii* complex bacteremia had a significantly lower survival rate than those with non-MDR *A*. *baumannii* complex bacteremia. Older age, being post-transplantation, higher Pittsburgh bacteremia score, and lower level of albumin were identified as independent risk factors for 30-day mortality in patients with MDR *A*. *baumannii* complex bacteremia.

Because the MDR rate among *A*. *baumannii* complex bacteremia varied by geographic area, patient age, setting of acquisition, and broad spectrum antibiotics exposure, regular surveillance of its changing trends is of particular importance. According to a retrospective study conducted in Thailand from 2005 to 2007, MDR *A*. *baumannii* complex bacteremia accounted for 49% of *A*. *baumannii* complex bacteremia [21]. In Korea from 2007 to 2010, carbapenem-resistant MDR *A*. *baumannii* complex bacteremia accounted for approximately 55.8% of all *A*. *baumannii* complex bacteremia episodes [22]. In our study, the overall MDR rate over five years of 75.52% of *A*. *baumannii* complex among cases of bacteremia was obviously higher than that in other countries. It is noteworthy that the proportion of MDR showed a downward trend from 2009 to 2010 and then grew steadily from 2010 to 2013. After reviewing the hospital management policies in West China Hospital, the drop in the drug-resistance rate from 2009 to 2010 may be due in large part to the rigid execution of infection control strategies during this period, which included routine patient based surveillance, strengthened environmental cleaning, strict contact isolation, promotion of hand hygiene, a multiple drug resistance screening program, and normalized antimicrobial prescribing policies [23]. With regard to the rise in the MDR rate among *A*. *baumannii* complex bacteremia from 2010 to 2013, there is no denying that the cumulative burden of prior use of broad spectrum antibiotics in critical patients and the rapid develop of drug resistance mechanisms in this specific pathogen play important roles [2].

The distribution of patients infected with *A*. *baumannii* complex and MDR *A*. *baumannii* complex concentrated in the ICU and surgical wards indicates that critical patients and post-surgical patients are more vulnerable to opportunistic pathogens due to suppressed immune states and their greater requirements for invasive devices.

Although they remain controversial in clinical research, polymyxins and tigecycline have almost always exhibited superiority to alternative antibiotics in *in vitro* studies of MDR *A*. *baumannii* complex [24]. As a result, the two antibiotics serve as the last defenders against these persistent bacteria in most clinical situations [1]. However, neither antibiotic is commercially available in most areas of mainland China. As a result, the combination therapy regimen, carbapenem plus sulbactam, is usually adopted in the face of MDR *A*. *baumannii* complex bacteremia. However, the effect of this combination therapy regimen is not ideal. Based on drug susceptibility results from our study, aminoglycosides or trimethoprim-sulfamethoxazole, which have relatively less resistance to MDR *A*. *baumannii* complex, may be explored in *in vitro* or *in vivo* studies for use in combination with other antibiotics to combat MDR *A*. *baumannii* complex in our area.

As MDR *A*. *baumannii* complex bacteremia usually leads to worse outcomes, factors related to MDR acquisition in these bacteria should be identified. According to several similar studies conducted in different regions, the risk factors for MDR acquisition in *A*. *baumannii* complex bacteremia may include the severity of the host’s condition, antimicrobial consumption, and infection control practices [25, 26]. The present study showed exposure to carbapenem and previous ICU admission to be independent risk factors. In addition to more severe health conditions in patients in ICU settings, cross-contamination, to a large extent, is associated with the high drug resistant rate, which has been demonstrated by previous molecular epidemiology research and numerous outbreaks of multidrug-resistant isolates in critical centers [21, 27]. Accordingly, to avoid the transmission of environmental organisms to patients from contaminated hands of medical staffs and equipment, it is of great necessity and importance to reinforce the infection control strategies, especially in the ICU. Meanwhile, we cannot ignore the impact of carbapenem abuse in the acquisition of drug resistance. Consumption of antibiotics has already been shown to be well correlated with increased microbial resistance [28–32]. Several previous studies focusing on carbapenem-resistant *A*. *baumannii* complex have shown that prior exposure to carbapenem increases the risk of acquiring carbapenem-resistant *A*. *baumannii* complex infection, which is consistent with our findings. Carbapenem-resistant *A*. *baumannii* complex accounted for approximately 91.8% of MDR *A*. *baumannii* complex in our present study. Based on the above, judicious prescribing of carbapenem with strict antibiotic stewardship is critical for controlling MDR *A*. *baumannii* complex infections. However, to delineate the specific drug resistance mechanism, such as production of other beta-lactamases, down-regulation of porins, enhanced efflux pump function, or formation of biofilms, is beyond the scope of this study [33, 34].

In this study, patients with MDR *A*. *baumannii* complex bacteremia also had increased 30-day mortality rates, which means that those patients tended to have worse prognoses. These findings are in line with those of earlier studies [35–37]. Lee et al. conducted a retrospective study of the clinical and economic impact of multidrug resistance in nosocomial *A*. *baumannii* bacteremia [38]. They reported that MDR *A*. *baumannii* bacteremia was associated with an excess attributable mortality rate of 21.8%, an excess length of hospital stay of 13.4 days, and excess hospital costs of $3,758, compared with non-MDR *A*. *baumannii* bacteremia. Similarly, a recent study in Thailand also demonstrated that the overall mortality rate was significantly higher in the group with MDR *A*. *baumannii* bacteremia than in the susceptible group (91.7% vs. 48%) [21]. It is now speculated that the greater probability of inappropriate empirical antibiotic therapy and the delay in the administration of appropriate antibiotic therapy, rather than the enhanced virulence of this pathogen, has resulted in more severe conditions and worse prognosis for patients with MDR *A*. *baumannii* complex bacteremia [39,40].

Our patients with MDR *A*. *baumannii* complex bacteremia had a 30-day mortality rate of 29.1%, which was similar to that in previous studies [41]. Such high mortality rates of MDR *A*. *baumannii* complex bacteremia were shown to be related to patients’ co-morbidity, sources of bacteremia, bacteremia severity, and inappropriateness of empirical therapy [42]. Our present study identified four independent factors affecting mortality, including older age, higher Pittsburgh score, lower serum albumin level and being post-transplantation. Pittsburgh score, a grading system consisting of five items (mental status, fever, hypertension, mechanical ventilation and cardiac arrest), was used to assess the severity of illness, and this has previously been shown to be highly predictive of prognosis of bacteremia caused by Pseudomonas and Enterobacter [43,44]. In our results, this tool also exhibited excellent performance in evaluating the outcome of MDR *A*. *baumannii* complex bacteremia, and the subsequent survival analysis also confirmed this conclusion. As one of the negative acute phase proteins, the level of albumin in the plasma of critical patients likely reflects the change in vascular permeability rather than changes in the synthesis or catabolism of the protein [45]. Thus, although albumin is traditionally regarded as an indication of nutritional status, it is not validated as a marker of the need to initiate nutrition support therapy in critical care patients [45]. However, our findings that lower level of albumin was an independent risk factor for 30-day mortality in patients with MDR *A*. *baumannii* complex support the theory that albumin could be a good indicator of prognosis in critically ill patients, which is consistent with previous reports [46]. In addition, patients who are post-transplantation had reduced survival rates in this study. With immunocompromised status, these patients were unable to control the infection effectively, which often leads to poor prognosis. Thus, to improve the cure rate, it is necessary to have heightened awareness of mortality risk in patients who are post-transplantation and to adopt potent antibiotics as soon as possible. It is also worth mentioning that a recently published study reported that polymicrobial bloodstream infections were significantly associated with a higher severity of illness, a longer duration of septic symptoms and a higher in-hospital mortality rate than monomicrobial bloodstream infections [47]. However, in our present research, the prognosis of patients with polymicrobial MDR *A*. *baumannii* complex bacteremia did not differ from the prognosis of patients with monomicrobial MDR *A*. *baumannii* complex bacteremia. One possible reason is that MDR *A*. *baumannii* complex may be the predominant determinant of prognosis compared with other organisms in polymicrobial bacteremia.

Our study should be interpreted with caution given its several limitations. First, the retrospective design may preclude the validity and reliability of comparisons. To diminish potential biases, multivariate logistic analysis was employed to discover independent risk factors. Additionally, all the data included in our analysis were retrieved from computerized records in order to reduce non-systematic information bias. Second, our data from a single center also limit the applicability of our results to other geographical areas or institutions. Third, as molecular studies cannot be performed because most clinical isolates in our center are not routinely preserved, resistance mechanisms of specific bacteria were not identified, and molecular epidemiology was not investigated. Lastly, our research focused on investigating the factors associated with the acquisition of MDR among *A*. *baumannii* complex bacteremia. However, the decision for empiric therapy against MDR *A*. *baumannii* infection needs to identify the risk factors of both MDR acquisition and *A*. *baumannii* complex bacteremia. In that situation, a case-case control study might be more effective in guiding clinical practice [11].

## Conclusion

In conclusion, our research revealed that exposure to carbapenem and previous ICU admission were independent risk factors for acquiring MDR among *A*. *baumannii* complex bacteremia. Patients with MDR *A*. *baumannii* complex bacteremia had worse outcomes, and their mortality risk factors were confirmed to be associated with older age, severity of illness and their immune status. The risk factors associated with acquisition of and mortality from MDR *A*. *baumannii* complex bacteremia may be used to prioritize infection control practice and prognosis evaluation.

## Supporting Information

S1 Table Antibiotics resistant rates of the *A*. *baumannii* complex isolates between MDR group and non-MDR group. (DOC)

S2 Table Comparison of demographics, clinical characteristics, infection status, and outcome of patients with MDR *A*. *baumannii* complex bacteremia and non-MDR *A*. *baumannii* complex bacteremia using an univariate model. (DOC)

S3 Table Comparison of risk factors associated with mortality in MDR *A*. *baumannii* complex bacteremia using an univariate model. (DOC)
