# Supplementary material for: Risk and Prognostic Factors for Multidrug-Resistant Acinetobacter Baumannii Complex Bacteremia: A Retrospective Study in a Tertiary Hospital of West China
Source: PLoS One. 2015 Jun 17;10(6):e0130701. doi: 10.1371/journal.pone.0130701 (PMC4471170; doi:10.1371/journal.pone.0130701)
Supplement: S1 Table — (DOC) [file pone.0130701.s001.doc]

**S1 Table. Antibiotics resistant rates of the *A. baumannii* complex isolates between MDR group and non-MDR group**

| **Antibiotics** | **MDR *A. baumannii* complex isolates (%)** | **Non-MDR *A. baumannii* complex isolates (%)** |
| --- | --- | --- |
| **GEN** | 174/182 (95.6) | 3/59 (5.1) |
| **AMK** | 78/150 (52.0) | 1/50 (2.0) |
| **TOB** | 159/182 (87.4) | 3/59 (5.1) |
| **IPM** | 167/182 (91.8) | 2/58 (3.4) |
| **LVX** | 169/182 (92.9) | 1/59 (1.7) |
| **CIP** | 176/182 (96.7) | 4/59 (6.8) |
| **TZP** | 125/127 (98.4) | 3/37 (8.1) |
| **TCC** | 54/55 (98.2) | 7/22 (31.8) |
| **CAZ** | 175/181 (96.7) | 7/59 (11.9) |
| **CTX** | 54/55 (98.2) | 13/21 (61.9) |
| **CRO** | 180/182 (98.9) | 42/59 (71.2) |
| **FEP** | 179/182 (98.4) | 9/59 (15.3) |
| **SXT** | 124/179 (69.3) | 12/59 (20.3) |
| **SAM** | 172/181 (95.0) | 11/58 (19.0) |
| **ATM** | 180/181 (99.4) | 54/58 (93.1) |

MDR, multidrug resistant; GEN, gentamicin; AMK, amikacin; TOB, tobramycin; IPM, imipenem; LVX, levofloxacin; CIP, ciprofloxacin; TZP, piperacillin/tazobactam; TCC, ticarcillin/clavulanic acid; CAZ, ceftazidime; CTX, cefotaxime; CRO, ceftriaxone; FEP, cefepime; SXT, trimethoprim-sulfamethoxazole; SAM, ampicillin/sulbactam; ATM, aztreonam.
