# Supplementary material for: Risk and Prognostic Factors for Multidrug-Resistant Acinetobacter Baumannii Complex Bacteremia: A Retrospective Study in a Tertiary Hospital of West China
Source: PLoS One. 2015 Jun 17;10(6):e0130701. doi: 10.1371/journal.pone.0130701 (PMC4471170; doi:10.1371/journal.pone.0130701)
Supplement: S2 Table — (DOC) [file pone.0130701.s002.doc]

**S2 Table. Comparison of demographics, clinical characteristics, infection status, and outcome of patients with MDR *A. baumannii* complex bacteremia and non-MDR *A. baumannii* complex bacteremia using an univariate model**

| **Variables** | **MDR *A. baumannii* complex** | **Non-MDR *A. baumannii* complex** | **Univariate analysis** |
| --- | --- | --- | --- |
|  | n=182 | n=59 | *p*-value |
| **Age, mean±SD (y)** | 52.423±18.507 | 58.678±15.117 | 0.021 |
| **Male, n (%)** | 126 (69.2%) | 41 (69.5%) | 0.970 |
| **BMI** | 22.865±3.511 | 22.968±3.870 | 0.909 |
| **Underlying disease, n (%)** |  |  |  |
| **Diabetes mellitus** | 22 (12.1%) | 8 (13.6%) | 0.766 |
| **Hypertension** | 46 (25.3%) | 14 (23.7%) | 0.811 |
| **Coronary artery disease** | 6 (3.2%) | 4 (6.8%) | 0.254 |
| **Chronic pulmonary disease** | 22 (12.1%) | 6 (10.2%) | 0.690 |
| **Cerebral vascular accident** | 13 (7.1%) | 4 (6.8%) | 0.925 |
| **Gastrointestinal disease** | 2 (1.1%) | 1 (1.7%) | 0.722 |
| **Hepatobiliary disease** | 10 (5.5%) | 5 (8.5%) | 0.414 |
| **Solid-organ malignancy** | 20 (11.0%) | 13 (22.0%) | 0.031 |
| **Hematology malignancy** | 9 (4.9%) | 1 (1.7%) | 0.300 |
| **End-stage Renal disease** | 14 (7.7%) | 4 (6.8%) | 0.817 |
| **Post-transplantation** | 8 (4.4%) | 0 (0.0%) | 0.995 |
| **Autoimmune disease** | 6 (3.3%) | 1 (1.7%) | 0.532 |
| **Primary admission diagnosis, n (%)** |  |  |  |
| **Respiratory infection** | 22 (12.1%) | 6 (10.2%) | 0.690 |
| **Traumatic injury** | 16 (8.8%) | 4 (6.8%) | 0.627 |
| **Acute pancreatitis** | 41 (22.5%) | 6 (10.2%) | 0.028 |
| **Selective operation** | 0 (0.0%) | 5 (8.5%) | 0.993 |
| **Hematology malignancy** | 8 (4.4%) | 1 (1.7%) | 0.360 |
| **Sources of bacteremia, n (%)** |  |  |  |
| **Respiratory infection** | 130 (71.4%) | 21 (35.6%) | <0.001 |
| **Urinary tract infection** | 8 (4.4%) | 5 (8.5%) | 0.236 |
| **Biliary tract infection** | 2 (1.1%) | 1 (1.7%) | 0.722 |
| **Central venous catheter infection** | 25 (13.7%) | 4 (6.8%) | 0.162 |
| **Post-surgical wound infection** | 12 (6.6%) | 0 (0.0%) | 0.042 |
| **Central nervous system infection** | 12 (6.6%) | 1 (1.7%) | 0.180 |
| **Intra-abdominal infection** | 26 (14.3%) | 6 (10.2%) | 0.420 |
| **Other** | 96 (52.7%) | 34 (57.6%) | 0.514 |
| **Severity of illness, n (%)** |  |  |  |
| **Pittsburgh score≥4** | 59 (32.4%) | 2 (3.4%) | <0.001 |
| **Interval between hospital admission and the sample date of first positive blood culture for AB (day)** | 19.725±27.716 | 20.797±41.634 | 0.821 |
| **Interval between ICU admission and the sample date of first positive blood culture for AB (day)** | 9.495±16.729 | 11.492±42.359 | 0.603 |
| **Invasive therapy within the past 14 days, n (%)** | 118 (64.8%) | 14 (23.7%) | 0.696 |
| **Presence of invasive devices, n (%)** | 167 (91.8%) | 38 (64.4%) | 0.009 |
| **Surgical procedure within the past 14 days, n (%)** | 77 (42.3%) | 22 (37.3%) | 0.496 |
| **Hospitalization within the past three months, n (%)** | 111 (61.0%) | 21 (35.6%) | <0.001 |
| **Stay in ICU, n (%)** | 109 (59.9%) | 7 (11.9%) | <0.001 |
| **Glucocorticoids/immunosuppressor use within the past 14 days, n (%)** | 74 (40.7%) | 6 (10.2%) | <0.001 |
| **Antibiotic use within the past 14 days, n (%)** |  |  |  |
| **Third-generation cephalosporins** | 17 (9.3%) | 7 (11.9%) | 0.575 |
| **Piperacillin/tazobactam** | 51 (28.0%) | 6 (10.2%) | 0.007 |
| **Cefoperazone/sulbactam** | 47 (25.8%) | 6 (10.2%) | 0.015 |
| **Carbapenems** | 88 (48.4%) | 2 (3.4%) | <0.001 |
| **Aztreonam** | 6 (3.3%) | 0 (0.0%) | 0.993 |
| **Floroquinolones** | 43 (23.6%) | 5 (8.5%) | 0.016 |
| **Macrolides/tetracyclines** | 3 (1.6%) | 0 (0.0%) | 0.995 |
| **Aminoglycoside** | 6 (3.3%) | 1 (1.7%) | 0.532 |
| **Vancomycin** | 44 (24.2%) | 2 (3.4%) | 0.003 |
| **Anti-fungal agents** | 45 (24.7%) | 3 (5.1%) | 0.003 |
| **Polymicrobial bacteremia, n (%)** | 139 (76.4%) | 23 (39.0%) | 0.155 |

MDR, multidrug-resistant; SD, standard deviation; OR, odds ratio; CI, confidence interval; BMI, body mass index; ICU, intensive care unit
