# Supplementary material for: Risk and Prognostic Factors for Multidrug-Resistant Acinetobacter Baumannii Complex Bacteremia: A Retrospective Study in a Tertiary Hospital of West China
Source: PLoS One. 2015 Jun 17;10(6):e0130701. doi: 10.1371/journal.pone.0130701 (PMC4471170; doi:10.1371/journal.pone.0130701)
Supplement: S3 Table — (DOC) [file pone.0130701.s003.doc]

**S3 Table. Comparison of risk factors associated with mortality in MDR *A. baumannii* complex bacteremia using an univariate model**

| **Variables** | **Mortality** | **Survival** | **Univariate analysis** |
| --- | --- | --- | --- |
|  | n=53 | n=129 | *p*-value |
| **Age, mean±SD (y)** | 56.434±18.510 | 50.775±18.324 | 0.062 |
| **Male, n (%)** | 35 (66.0%) | 91 (70.5%) | 0.550 |
| **BMI** | 23.062±3.615 | 22.772±3.506 | 0.696 |
| **Underlying disease, n (%)** |  |  |  |
| **Diabetes mellitus** | 5 (9.4%) | 17 (13.2%) | 0.483 |
| **Hypertension** | 13 (24.5%) | 33 (25.6%) | 0.882 |
| **Coronary artery disease** | 3 (5.7%) | 3 (2.3%) | 0.267 |
| **Chronic pulmonary disease** | 10 (18.9%) | 12 (9.3%) | 0.078 |
| **Cerebral vascular accident** | 5 (9.4%) | 8 (6.2%) | 0.445 |
| **Gastrointestinal disease** | 1 (1.9%) | 1 (0.8%) | 0.527 |
| **Hepatobiliary disease** | 4 (7.5%) | 6 (4.7%) | 0.440 |
| **Solid-organ malignancy** | 8 (15.1%) | 12 (9.3%) | 0.261 |
| **Hematology malignancy** | 4 (7.5%) | 5 (3.9%) | 0.308 |
| **End-stage Renal disease** | 6 (11.3%) | 8 (6.2%) | 0.246 |
| **Post-transplantation** | 5 (9.4%) | 3 (2.3%) | 0.049 |
| **Autoimmune disease** | 2 (3.8%) | 4 (3.1%) | 0.818 |
| **Other** |  |  |  |
| **Blood culture detection time (day)** | 4.248±1.256 | 4.264±0.880 | 0.932 |
| **Imipenem resistant, n (%)** | 50 (94.3%) | 117 (90.7%) | 0.827 |
| **Primary admission diagnosis, n (%)** |  |  |  |
| **Respiratory infection** | 8 (15.1%) | 14 (10.9%) | 0.427 |
| **Traumatic injury** | 4 (7.5%) | 12 (9.3%) | 0.705 |
| **Acute pancreatitis** | 6 (11.3%) | 35 (27.1%) | 0.025 |
| **Selective operation** | 0 (0.0%) | 0 (0.0%) | - |
| **Hematology malignancy** | 3 (5.7%) | 5 (3.9%) | 0.596 |
| **Other** | 32 (60.4%) | 64 (49.6%) | 0.188 |
| **Sources of bacteremia, n (%)** |  |  |  |
| **Respiratory infection** | 41 (77.4%) | 89 (69.0%) | 0.258 |
| **Urinary tract infection** | 0 (0.0%) | 8 (6.2%) | 0.995 |
| **Biliary tract infection** | 1 (1.9%) | 1 (0.7%) | 0.527 |
| **Central venous catheter infection** | 6 (11.3%) | 19 (14.7%) | 0.545 |
| **Post-surgical wound infection** | 2 (3.8%) | 10 (7.8%) | 0.336 |
| **Central nervous system infection** | 3 (5.7%) | 9 (7.0%) | 0.746 |
| **Intra-abdominal infection** | 7 (13.2%) | 19 (14.7%) | 0.790 |
| **Severity of illness, n (%)** |  |  |  |
| **Pittsburgh bacteremia score≥4** | 23 (43.4%) | 36 (27.9%) | 0.044 |
| **Interval between hospital admission and the sample date of first positive blood culture for AB (day)** | 19.623±17.922 | 19.767±30.916 | 0.974 |
| **Interval between ICU admission and the sample date of first positive blood culture for AB (day)** | 7.830±11.662 | 10.178±18.408 | 0.398 |
| **Invasive therapy within the past 15 days, n (%)** | 14 (26.4%) | 88 (68.2%) | 0.602 |
| **Stay of ICU, n (%)** | 35 (66.0%) | 74 (57.4%) | 0.453 |
| **Polymicrobial bacteremia, n (%)** | 28 (52.8%) | 99 (76.7%) | 0.254 |
| **Gram-positive bacteria** | 15 (28.3%) | 89 (69.0%) | 0.405 |
| ***Staphylococcus aureus*** | 0 (0.0%) | 3 (2.3%) | 0.995 |
| **Coagulase negative *staphylococcus*** | 1 (1.9%) | 4 (3.1%) | 0.652 |
| ***Enterococcus faecalis*** | 2 (3.8%) | 4 (3.1%) | 0.818 |
| ***Enterococcus faecium*** | 8 (15.1%) | 9 (7.0%) | 0.095 |
| **Gram-negative bacteria** | 15 (28.3%) | 88 (68.2%) | 0.579 |
| ***Enterobacter*** | 9 (17.0%) | 18 (14.0%) | 0.602 |
| ***Acinetobacter* excluding *Acinetobacter baumannii*** | 0 (0.0%) | 0 (0.0%) | - |
| ***Pseudomonas*** | 2 (3.8%) | 5 (3.9%) | 0.974 |
| **Candida** | 3 (5.7%) | 7 (5.4%) | 0.950 |
| ***Candida albicans*** | 1 (1.9%) | 4 (3.1%) | 0.652 |
| ***Candida glabrata*** | 0 (0.0%) | 1 (0.8%) | 0.995 |
| ***Candida tropicalis*** | 2 (3.8%) | 2 (1.6%) | 0.368 |
| ***Candida parapsilosis*** | 0 (0.0%) | 0 (0.0%) | - |
| ***Candida krusei*** | 1 (1.9%) | 0 (0.0%) | 0.993 |
| **Serum albumin (g/L)** | 28.059±7.569 | 31.953±12.063 | 0.009 |

MDR, multidrug-resistant; SD, standard deviation; OR, odds ratio; CI, confidence interval; BMI, body mass index; ICU, intensive care unit
